# A Meta-Analysis of the Therapeutic Effects of Glucagon-Like Peptide-1 Agonist in Heart Failure

**DOI:** 10.1155/2012/249827

**Published:** 2012-07-01

**Authors:** Mohammed Munaf, Pierpaolo Pellicori, Victoria Allgar, Kenneth Wong

**Affiliations:** Department of Cardiovascular and Respiratory Studies, Hull and East Yorkshire Medical Research and Teaching Centre, Daisy Building, Castle Hill Hospital, Castle Road, Kingston upon Hull HU16 5JQ, UK

## Abstract

We conducted a meta-analysis of the existing literature of the therapeutic effects of using GLP-1 agonists to improve the metabolism of the failing heart. Animal studies showed significant improvement in markers of cardiac function, such as left ventricular ejection fraction (LVEF), with regular GLP-1 agonist infusions. In clinical trials, the potential effects of GLP-1 agonists in improving cardiac function were modest: LVEF improved by 4.4% compared to placebo (95% C.I 1.36–7.44, *P* = 0.005). However, BNP levels were not significantly altered by GLP-1 agonists in heart failure. In two trials, a modest increase in heart rate by up to 7 beats per minute was noted, but meta-analysis demonstrated this was not significant statistically. The small number of studies plus variation in the concentration and length of the regime between the trials would limit our conclusions, even though statistically, heterogeneity chi-squared tests did not reveal any significant heterogeneity in the endpoints tested. Moreover, studies in non-diabetics with heart failure yielded conflicting results. In conclusion, the use of GLP-1 agonists has at best a modest effect on ejection fraction improvement in heart failure, but there was no significant improvement in BNP levels in the meta-analysis.

## 1. Introduction

Heart failure (HF) is defined as “*a complex clinical syndrome that can result from any structural or functional cardiac disorder that impairs the ability of the ventricle to fill with or eject blood*” [1]. HF is a major public health issue, with a prevalence of over 5.8 million in the USA, and over 23 million (and rising) worldwide. The lifetime risk of developing HF is one in five [2]. Despite advances in treatment, the number of deaths from heart failure has increased steadily and only one quarter to one-third of people with heart failure survive 5 years after admission [3]. The cause of heart failure has shifted in the last two decades: in the late 1970s, rheumatic valvular disease was the primary cause, nowadays the leading cause is ischemic heart disease [4]. A deficit in the “pump” function as cause of signs or symptoms attributed to HF, or systolic dysfunction, is frequently well diagnosed due to widespread availability of echocardiography but, an increased left ventricular (LV) “stiffness,” or diastolic dysfunction, is often missed. To further complicate matters, the two components—systolic and diastolic dysfunction—often coexist. Some studies [5, 6] reported that isolated diastolic dysfunction could be responsible for up to 50% of heart failure admissions (often labelled as “heart failure with normal ejection fraction,” HFnEF), with a major impact on patient outcome. Moreover, in patients with impaired glucose tolerance, the extent of diastolic dysfunction seems to be more severe [7] and HFnEF seems to be more common in patients with a history of hypertension and/or diabetes [8, 9].

The standard treatment of systolic heart failure is currently angiotensin-converting enzyme (ACE) inhibitors, angiotensin II receptor blockers (ARBs), beta blockers, and aldosterone antagonists. These all improve prognosis of heart failure. However, there is no specific treatment for HFnEF: diuretics are often used for symptom control; digoxin is particularly beneficial for ventricular rate control when atrial fibrillation (AF) is the predominant rhythm.

In recent years, progress in basic research has led to the identification of multiple new possible therapeutic targets for the treatment of systolic heart failure, and many promising drugs have subsequently been developed. These include novel vasodilators, such as natriuretic peptides, metabolic substrates, urocortins, guanylyl cyclase activators, and adrenomedullin. They also include drugs such as direct renin inhibitors, and aldosterone synthase inhibitors [10]. There have been numerous large randomised controlled trials (RCT) of these new drugs. They have not yet been licensed as results regarding the efficacy of these new drugs have not been entirely positive. Further evidence is needed as many of the positive results that have been observed in preclinical studies and Phase II trials have not always been confirmed in Phase III studies [10].

As mentioned above, the leading cause of systolic HF is myocardial ischaemia, whereby the myocardium is oxygen starved and thus has a decreased ability to generate ATP by oxidative metabolism. As a result, it is unable to effectively transfer the chemical energy from the metabolism of carbon fuels to contractile work. This leads the myocardium to utilise other compounds, such as free fatty acids (FFAs), for energy production. However, if the heart uses FFAs as a substrate for energy generation, there is much greater oxygen consumption per unit ATP produced than there is with glucose. This increased demand for oxygen can lead to worsening heart failure. Thus, improvement of cardiac energetics is an important therapeutic target in patients with heart failure [10].

Metabolic modulators do exactly this by altering the substrate that is oxidized by the myocardium to derive energy. They shift this substrate from FFA to glucose and thus optimize metabolic efficiency of the heart. These compounds exert their effects through several mechanisms: inhibiting carnitine *O*-palmitoyltransferase 1, long-chain 3-ketoacyl-CoA thiolase or malonyl-CoA decarboxylase, reducing plasma levels of FFA and myocardial uptake of FFA, and/or activating the 5′-AMP-activated protein kinase (AMPK). Thus it follows that, using metabolic manipulating agents to either promote glucose utilisation or reduce fatty acid utilisation, will improve the metabolic efficiency of the heart by decreasing oxygen demand and thus be used therapeutically in heart failure. Amongst these metabolic agents are glucagon like peptide-1 (GLP-1) agonists [10].

GLP-1 is an incretin that is released from intestinal L cells in response to glucose ingestion and is known to be a potent glucose-dependent insulinotropic hormone. It has important actions on gastric motility, on the suppression of plasma glucagon levels, and possibly on the promotion of satiety and stimulation of glucose disposal in peripheral tissues independent of the actions of insulin. It does this by increasing insulin secretion from the pancreas and myocardial glucose uptake via the translocation of glucose-transporting vesicles (glucose transporter type 1 (GLUT1) and GLUT4) to the sarcolemma. GLP-1 exerts its direct cardioprotective effects through the stimulation of G-protein-coupled receptors (i.e., GLP1Rs) that are coupled to adenylyl cyclase, and via its rapid metabolism to the GLP1 (9–36) amide [11].

Therefore, GLP-1 agonists can be used to bring about the same effects. These agents have been investigated widely as an adjunct to therapy in diabetes as they offer an obvious alternative to insulin, but their metabolic effect could also be extended to the heart as they can enable the heart to switch to the more energy-efficient glucose-dependent pathway [10]. Moreover, there are GLP-1 specific receptors in cardiac tissue so the potential for using these peptide agonists holds promise for treating heart failure [12].

However, whilst GLP-1-related compounds have proven efficacy in the treatment of hyperglycaemia associated with type 2 diabetes [13, 14], little was known about the effectiveness of GLP-1 agonist or other peptides substrates in improving cardiac function in heart failure. Because the half-life of GLP-1 in only a few minutes, several Phase III-Phase IV trials are analysing the effects of its analogues, such as exenatide, which are not degraded so quickly [15].

## 2. Aims and Objectives

We aimed to carry out a comprehensive review of medical literature on the therapeutic advantage of using peptide agonists to improve cardiac metabolism in heart failure. We included all papers regardless of size, whether they were pre-clinical or clinical trials, either randomized, blinded, or not. The results of these papers have been combined to give an overall estimate of the effectiveness of using GLP-1 agonists in heart failure. Furthermore, we conducted a meta-analysis of each primary outcome if contained in more than two papers.

## 3. Methods

### 3.1. Search Strategy of the Meta-Analysis

Highly sensitive search strategies were developed using appropriate subject headings and text word terms. Full details of the search strategies used are appended. The following electronic databases were searched: the Cochrane Library (Issue 7, 2011); MEDLINE (via OVID, from 1948 to August week 1 2011); Pubmed (via NCBI); EMBASE (via OVID, from 1996 to week 30, 2011); BMJ's Clinical Evidence; DARE (Issue 7, 2011). British and American medical journals were also hand-searched, such as The Lancet, NEJM, and BMJ. In addition, conference proceedings and reference lists of all included studies were scanned to identify additionally potentially relevant studies. There were no start year or language restrictions.

### 3.2. Data Extraction

One reviewer screened the titles (and abstracts if available) of all reports identified by the search strategy. Full copies of potentially relevant reports were obtained, studied, and assessed for inclusion. Data was discussed with the senior author, and disagreements were resolved by consensus.

### 3.3. Selection Criteria

Papers that had details of trials conducted of peptide agonists versus placebo or usual treatment alone for heart failure were included. All papers, whether they included human or animal trials were included. For humans, randomized controlled trials, regardless of whether they were blinded, were included along with pilot and observational studies.

### 3.4. Meta-Analysis Methodology

#### 3.4.1. Data Synthesis

The eligible trials were entered into RevMan 5 software package, and the statistical methods were those programmed into RevMan 5.1 analysis software.

For continuous data, the mean difference and 95% confidence intervals were calculated. Where applicable, for dichotomous data, the relative risk and 95% confidence intervals would be calculated. The results from the trials were pooled using the fixed effects models. We tested for heterogeneity with the chi squared statistic, which was considered to be significant at *P* < 0.10. If significant, a random effect model would be used to allow generalisation of the results and sources of heterogeneity would be investigated. *Z* tests were used to test for the overall effect.

## 4. Results

A total of 16 papers were found in Medline and 32 in Embase. Handsearching in Pubmed yielded a further 22 papers. There were no Cochrane or DARE reviews of the use of GLP-1 agonist due to the scarcity of clinical trials on these agents and there were no additional papers found in American or British journals. The full references of the papers which contained studies are listed below in the references section.

The general finding from Medline, Embase, and Pubmed was that the papers that were found to mention GLP-1 agonists in HF, generally only detailed their pharmacology and suggested their potential for therapeutic benefit with very few containing any experimental evidence for the application of these agents [10–23]. When these papers containing studies were examined, they pertained to the use of GLP-1 agonists in diabetics with HF due to their insulinotropic effects instead of looking at their use as metabolic substrates for the ischaemic heart as has been suggested by some other papers. In the present paper, we only focused on papers that had experimental evidence for the use of GLP-1 agonists as therapeutic agents. These are discussed below.

### 4.1. Preclinical Experiments

Work on rats [24, 25], rabbits [26], mice [27], and dogs [28, 29] showed favourable functional effects of GLP-1 in failing hearts with significant improvements in LV systolic and diastolic function.

Nikolaidis et al. [28] found that short-term infusion of recombinant GLP-1 over 48 hours increased myocardial insulin sensitivity and glucose uptake in a canine model of rapid pacing-induced dilated cardiomyopathy. Interestingly, GLP-1 (9–36) was found to exert similar beneficial effects to native GLP-1 in this model, supporting the growing suggestion that the metabolically inactive form of GLP-1 [GLP-1 (9–36)] may play an active role in the cardiovascular system.

Furthermore, spontaneously hypertensive heart-failure-prone rats (characterized by obesity, insulin resistance, hypertension, and dilated cardiomyopathy), treated chronically with GLP-1 from 9 months of age (when they begin to progress to advanced heart failure and death) exhibited preserved cardiac contractile function, increased myocardial glucose uptake, improved survival, and a significant reduction in cardiac myocyte apoptosis [22]. Although this study also reported GLP-1 to stimulate myocardial glucose uptake in the failing myocardium, it was unclear whether its beneficial effects on contractile function occurred due to a direct cardiac action or was secondary to its established insulinotropic effects. These promising findings led the way for clinical trials and these are discussed below.

### 4.2. Clinical Trials

The beneficial effects on contractile function seen in animals treated with GLP-1 were supported by preliminary clinical studies in humans, indicating that GLP-1 may also improve LV contractile function in patients with chronic heart failure.

Thrainsdottir et al. [30], in an early nonrandomised pilot investigation conducted on 6 hospitalised type 2 diabetic hospitalised with ischaemic but stable heart failure New York Heart Association (NYHA) class II-III, with LVEF < 40%, found that short-term GLP-1 infusion for 3 days tended to improve both systolic and diastolic function, although these changes did not reach statistical significance.

However, we also found another three-day study that was conducted on 10 patients with acute myocardial infarction (AMI) or left ventricular ejection fraction (LVEF) of <40% compared with 11 controls [20]. Baseline demographics and background therapy were similar, and both groups had severe LV dysfunction at baseline (LVEF = 29 ± 2%). The study demonstrated that GLP-1 significantly improved LVEF (from 29 ± 2% to 39 ± 2%, *P* ≤ 0.01), global wall motion score indexes (1.94 ± 0.11 → 1.63 ± 0.09, *P* ≤ 0.01), and regional wall motion score indexes (2.53 ± 0.08 → 2.02 ± 0.11, *P* ≤ 0.01) compared with control subjects. The benefits of GLP-1 were independent of AMI location or history of diabetes. Moreover, GLP-1 was well tolerated, with only transient gastrointestinal effects.

Moreover, longer-term treatment with GLP-1 has shown positive results in both diabetics and nondiabetics. Sokos and colleagues [31] compared a 5-week infusion of GLP-1 added to standard therapy in 12 patients with NYHA class III/IV heart failure and the results were compared with those of 9 patients with heart failure on standard therapy. They found that patients treated with GLP-1 infusion had significantly better LV systolic function (LVEF changed from 21 ± 3% to 27 ± 3%  *P* < 0.01), exercise tolerance (VO_2_ max changed from 10.8 ±  .9 mL/O_2_/min/kg to 13.9 ±  .6 mL/O_2_/min/kg; *P* < 0.001, as well as the 6-minute walk distance, from 232 ± 15 m to 286 ± 12 m; *P* < 0.001), and quality of life (Minnesota Living with Heart Failure quality of life score (MNQOL) score: from 64 ± 4 to 44 ± 5; *P* < 0.01). However, no significant changes in any of the parameters were observed in the control group on standard therapy. GLP-1 was well tolerated with minimal episodes of hypoglycaemia and gastrointestinal side effects. Like the aforementioned study [20], this study suggests a role for GLP-1 agonists beyond glycaemic control as significant improvements were seen in both diabetic and nondiabetic patients.

However, we found no further evidence for the extension of GLP-1 to nondiabetics. In a randomized, double-blind crossover trial of 20 normoglycaemic patients without diabetes and with HF with ischemic heart disease, severe left ventricular impairment, NYHA II, and III, Halbirk et al. [32] found that GLP-1 infusion over 48 h increased circulating insulin levels and reduced plasma glucose concentration but had no major cardiovascular effects in patients with chronic heart failure when compared with a placebo. The only significant cardiovascular impacts of the infusion were increases in heart rate (67 ± 2 beats/min versus 65 ± 2 beats/min; *P* = 0.016) and diastolic blood pressure (71 ± 2 mmHg versus 68 ± 2 mmHg; *P* = 0.008). GLP-1 had no effect on systolic blood pressure (113 ± 5 mmHg versus 113 ± 4 mmHg; *P* = 0.95) or on LVEF (GLP-1 treatment from 28 ± 2% to 30 ± 2% versus placebo 30 ± 2% to 30 ± 2%; *P* = 0.93). Importantly, also, GLP-1 infusion did not affect exercise capacity, VO_2_ max, cardiac index, stroke volume, and systemic vascular resistance during exercise. Unlike other studies, hypoglycemia was frequent with eight patients experiencing nine episodes of hypoglycaemia (capillary glucose < 3.5 mmol/L) versus none with placebo. This calls for caution in patients without diabetes but with HF and also reiterates the need for further studies with regard to the use of GLP-1 agonists in nondiabetics. Intriguingly, both GLP-1 and placebo significantly dropped BNP, although the effects of the two infusions did not differ (−112 ± 54 pg/mL versus −65 ± 54 pg/mL, *P* = 0.17). Future trials looking at changes in BNP in heart failure should bear in mind that small changes need to be interpreted with caution, as it was intriguing that placebo might have produced a significant reduction in BNP. The authors of that paper attributed this drop in natriuretic peptide to be due to patients' reduced exercise during their hospital stay, more than a direct effect of the infusion. However, a recent study conducted in healthy subjects found exenatide had significant haemodynamic effects, including natriuretic properties [33].

### 4.3. Meta-Analysis

**Figure 1:**
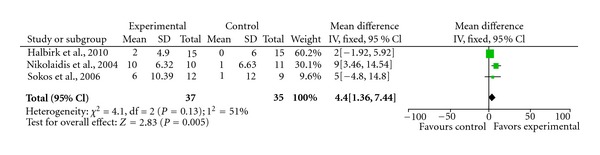
Forrest plot demonstrating GLP-1 improves ejection fraction.

**Figure 2:**
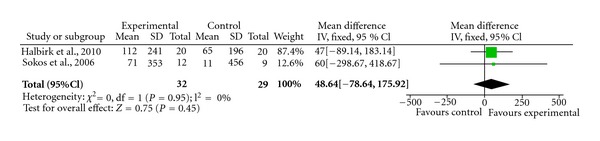
Forrest plot demonstrating the negligible effect of GLP-1 on BNP levels.

**Figure 3:**
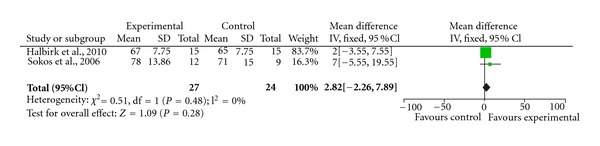
Forrest plot demonstrating the effect of GLP-1 agonist on heart rate.

Individually, some of the studies that we have discussed would suggest that GLP-1 agonist might be potentially effective for heart failure. We performed a meta-analysis on all the primary endpoints that were contained in at least two papers. The results were summarised in Table 1, and Figures 1, 2, and 3.

There was at best a modest improvement in ejection fraction (4.4%; 95% CI 1.36–7.44%). There was no significant change in BNP or heart rate in our meta-analysis. Thus, although some of the preliminary clinical studies provided some encouragement for the potential use of GLP-1 in the treatment of heart failure, it is clear that significant further research is required to confirm these initial observations, investigate the underlying mechanisms, and explore possible interactions with current heart failure therapies.

### 4.4. Limitations of Meta-Analysis

As with any meta-analysis, the quality is dependent on the quality of the studies and any limitations the included studies have. Firstly, the most obvious limitation is the lack of a large number of studies available to meta-analyse. Secondly, the total sample size of patients in all four studies combined is small. A further limitation in our meta-analysis is that all four studies investigated different concentrations of GLP-1 agonist infusion: 1.0 pmol/kg/min (Halbirk); 1.5 pmol/kg/min (Nikolaidis); 2.5 pmol/kg/min (Sokos) and 4 pmol/kg/min (Thrainsdottir). Moreover, the studies measured improvements at different intervals of time, with Halbirk looking at effects after 48 hours, Thrainsdottir and Nikkolaidis at 3 days and Sokos investigating a 5-week infusion. This has definite implications for interpretation of the results. Another limitation was that not all the studies included were double blinded and randomised, for example, Thrainsdottir was an open observation study, whereas Halbirk was a double-blinded crossover placebo study. This leads to methodological heterogeneity.

### 4.5. Clinical Implications and Future Research

The Carvedilol Hibernating Reversible Ischaemia Trial: Marker of Success (CHRISTMAS trial) [34] found patients with more hibernation/ischaemia had greater improvement in left ventricular systolic function with beta-blocker treatment. Our Academic Cardiology Department in Hull also conducted the Heart Failure Revascularisation Trial which showed how myocardial ischaemia and hibernation could not effectively be resuscitated by revascularization in patients with chronic HF [35]. Recently, the large STITCH trial [36] did not demonstrate any survival benefit of coronary artery bypass surgery in patients with heart failure with severe coronary artery disease. Thus, to optimally treat ischaemic heart failure, we need to explore other avenues to improve myocardial metabolism, to try and optimize cardiac function.

GLP-1 is an endogenous peptide which is released from the gut following food intake. It is one of a number of factors that can augment insulin release, so as expected, its role in improving glycaemic control in diabetics is now fairly well established.

Our meta-analysis of clinical trials involving patients with heart failure demonstrated some promising evidence to suggest possible beneficial effects of the GLP-1 peptide agonist in improving cardiac function, in both diabetics and nondiabetics. This was seen with the statistically significant increase in left ventricular ejection fraction, although the absolute change was very modest (4.4%). An absence of lowering effect on systolic blood pressure may be particularly appealing to clinicians who find their patients with heart failure often have relatively low blood pressure on a combination of ACE-inhibitors, beta blockers, spironolactone or eplerenone, and loop diuretics. It should be noted that the drug might drop patients' diastolic blood pressure.

Minor increase in heart rate may also turn out to be a concern as recent evidence have confirmed the hypothesis that patients with heart failure have better prognosis if their heart rate is less than 70 beats per minute [37]. However, whilst in the two individual trials (Halbirk and Sokos), there was a modest increase in heart rate by up to 7 beats per minute, our meta-analysis demonstrated this was not significant statistically. In nondiabetics with heart failure, caution must be exercised to ensure they do not develop hypoglycaemia, which again is potentially hazardous.

Before the peptide agonist can be recommended for routine clinical use, large multicentre, double-blinded randomised controlled trials are needed, investigating the effects of GLP-1 or its analogue in patients with acute or chronic HF including hard endpoints, such as mortality, cardiovascular death, or hospitalization for heart failure. Further, as suggested previously, heart failure with normal ejection fraction (HFnEF) is often difficult to treat specifically. Future trials should study the effect of GLP-1 agonists in this challenging group of patients. Recent work suggested that advanced echocardiography techniques using speckle tracking to assess the so-called global longitudinal strain (GLS) might identify patients with subtle systolic dysfunction [38] and might even be better than ejection fraction at predicting poor cardiovascular outcome in patients with chronic heart failure [39]. 

## 5. Conclusions

This meta-analysis of the potential therapeutic benefits of GLP-1 agonists in heart failure involved a thorough literature search using Embase and Medline plus hand-search strategies. The animal studies gave evidence in favour of these peptide agonists. There were only a few small clinical trials involving patients with heart failure. The use of GLP-1 agonists has at best a modest effect on ejection fraction improvement in patients with heart failure, but there was no significant improvement in BNP levels in the meta-analysis.

## Figures and Tables

**Figure 4:**
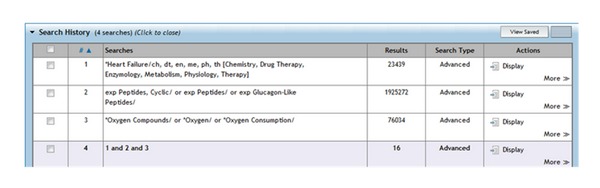
Full Medline search with MeSH terms.

**Figure 5:**
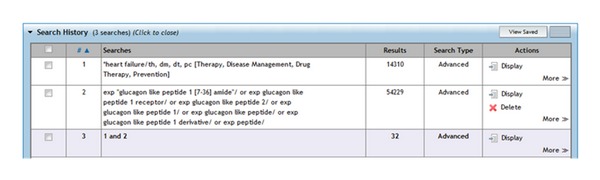
Full Embase search with MeSH terms.

**Table 1:** Summary of all trials studying GLP-1 effects in human heart failure.

Study	Endpoints
Thrainsdottir et al., 2004 [30]	HR, BP (rest + exercise), rate pressure product, global systolic and diastolic function, LVEF, LV end-diastolic diameter

Nikolaidis et al., 2004 [20]	LVEF, ED +ESV, SV, global WMSI

Sokos et al., 2006 [31]	HR, BNP, LVEF, VO_2_, 6-min walk

Halbirk et al., 2010 [32]	BNP, BP, HR, SV, CI, LVEF, SVR, 6 min hall walk test

## Appendix

Medline Search Strategy The search focused on heart failure, mapped to subject headings, and included the following MESH terms: chemistry, drug therapy, enzymology, metabolism, physiology, and therapy. The second search term was for peptide and this term was again mapped to include medical subject headings. From this the following mesh terms were exploded: Glucagon-like peptides; peptides and peptides, cyclic. The third search term was oxygen, including oxygen compounds, oxygen, and oxygen consumption as these were central to our review. The fourth search item combined the above three and produced 16 papers. The full search is shown in Figure 4.

Embase Search Strategy For Embase, again heart failure was the first search term, including disease management, drug therapy, prevention, and therapy. The second search term was for peptide and all the similar MeSH including “glucagon like peptides” were selected. All these were exploded so similar terms could be included. The third search combined the previous two searches with “AND,” thus returning 32 results: see Figure 5.

Hand-Searching Pubmed yielded a further 22 papers. Of these, only three papers contained results of studies done on humans. These, along with the papers found with Medline and Embase, were cited fully in the references section. There were no additional papers found in the medical journals that were hand-searched (BMJ, Lancet, NEJM).
